# Hyperbaric oxygen therapy as an adjunctive treatment for sternal infection and osteomyelitis after sternotomy and cardiothoracic surgery

**DOI:** 10.1186/1749-8090-6-141

**Published:** 2011-10-17

**Authors:** Wen-Kuang Yu, Yen-Wen Chen, Huei-Guan Shie, Te-Cheng Lien, Hsin-Kuo Kao, Jia-Horng Wang

**Affiliations:** 1 Department of Respiratory Therapy, Taipei Veterans General Hospital, Taipei, Taiwan; 2 National Yang-Ming University School of Medicine, Taipei, Taiwan

**Keywords:** hyperbaric oxygen, sternal infection, osteomyelitis, sternotomy, Cardiothoracic surgery

## Abstract

**Purpose:**

A retrospective study to evaluate the effect of hyperbaric oxygen (HBO2) therapy on sternal infection and osteomyelitis following median sternotomy.

**Materials and methods:**

A retrospective analysis of patients who received sternotomy and cardiothoracic surgery which developed sternal infection and osteomyelitis between 2002 and 2009. Twelve patients who received debridement and antibiotic treatment were selected, and six of them received additional HBO2 therapy. Demographic, clinical characteristics and outcome were compared between patients with and without HBO2 therapy.

**Results:**

HBO2 therapy did not cause any treatment-related complication in patients receiving this additional treatment. Comparisons of the data between two study groups revealed that the length of stay in ICU (8.7 ± 2.7 days vs. 48.8 ± 10.5 days, p < 0.05), duration of invasive (4 ± 1.5 days vs. 34.8 ± 8.3 days, p < 0.05) and non-invasive (4 ± 1.9 days vs. 22.3 ± 6.2 days, p < 0.05) positive pressure ventilation were all significantly lower in patients with additional HBO2 therapy, as compared to patients without HBO2 therapy. Hospital mortality was also significantly lower in patients who received HBO2 therapy (0 case vs. 3 cases, p < 0.05), as compared to patients without the HBO2 therapy.

**Conclusions:**

In addition to primary treatment with debridement and antibiotic use, HBO2 therapy may be used as an adjunctive and safe treatment to improve clinical outcomes in patients with sternal infection and osteomyelitis after sternotomy and cardiothoracic surgery.

## Introduction

Patients with sternal infection and osteomyelitis after sternotomy and cardiothoracic surgery are uncommon, but these serious complications could increase postoperative mortality, morbidity and medical cost [1,2]. Ischemia and hypoxia are postulated as the mechanism resulting in development of sternal infection and osteomyelitis [3,4]; however, the current primary treatment only focuses on early debridement and antibiotic use [5].

Hypobaric oxygen (HBO2) therapy, the administration of 100% oxygen at 2 to 3 absolute atmosphere pressure (ATA), has been widely used in the treatment of various problem wounds and for refractory osteomyelitis [6,7]. Mechanisms of HBO2 therapy include reversing hypoxia, reducing local edema, improving host immunity, enhancing antibiotic activity [8,9]. There are only few reports about the additional HBO2 therapy for patients who develop sternal osteomyelitis after sternotomy. Most of the results are effective and successful [10,11]. The aim of this retrospective study was to evaluate the efficacy of HBO2 therapy in patients with sternal infection and osteomyelitis after sternotomy and cardiothoracic surgery.

## Methods and materials

During January 1^st^, 2002 to December 31^th ^2009, twelve patients who developed sternal infection and osteomyelitis following sternotomy and cardiothoracic surgery at Taipei Veterans General Hospital were recruited. The study was approved by the Institutional Review Board of the Taipei Veterans General Hospital (approval number, 201009015IC). All study data were collected retrospectively, so informed consent was not required.

The diagnosis of organ/space sternal surgical site infection(SSI) was according to the Center for Disease Control (CDC) criteria: (1) the sternal SSI occurs after sternotomy and cardiothoracic surgery; (2) the infection appears to be related to the operation and could involve any part of the anatomy other than the incision which was opened or manipulated during the operation (organs or spaces). Furthermore, at least one of the following criteria is required: (1) purulent drainage from the organ/space of sternal surgical site; (2) organism isolated from an aseptically obtained culture of fluid or tissue in the organ/space of sternal surgical site; (3) an abscess or other evidence of infection involving the organ/space of sternal surgical site found on direct examination; (4) diagnosis of organ/space SSI by a surgeon or attending physician [12]. In addition, sternal osteomyelitis was documented by computer tomography of chest, surgical debridement pathology or osteomyelitis scans.

All patients were treated with empiric antibiotics initially and then guided by culture report and antibiotic susceptibility tests. Early debridement, daily antiseptic irrigation and dressing change were performed. After the infection was under control, reconstruction of the wound with pectoralis or rectus muscle and omental flap rotation was performed at the decision of the clinical physician. HBO2 therapy was performed in a multiplace chamber (HTK 1500/BS, Drägen, Siemens, Germany). Each session was 120 minutes long including three phases: compression, oxygen breath and decompression. Compression and decompression were performed with room air at a rate of 0.1 ATA per minute. At oxygen breath phase, all patients breathed 100% oxygen under 2.5 ATA for 90 minutes through a face mask that was fit well and secured with head straps. During the treatment period, patients were observed closely for acute illness or any complications. HBO2 therapy sessions were daily from Monday to Friday with a break of 2 days. The total number of sessions of HBO2 therapy performed based on clinical outcome and at the discretion of the clinical physician.

### Statistical analysis

Statistical analysis was done using SPSS version 18.0 (SPSS Inc., Chicago IL). Continuous variables were expressed as the mean ± standard deviation (SD). Continuous variables were compared with Wilcoxon's rank sume test and categorical variables were compared by Fishers exact test. *p *< 0.05 was considered statistically significant.

## Results

During the study period, 12 patients (mean age 59 ± 4.5 years) fulfilling the entry criteria were selected. Among these patients, 7 patients received coronary arterial bypass surgery, 3 patients received thymectomy, 1 patient received mitral valve replacement and 1 patient received type A aortic dissection repair. They received computer tomography of chest (n = 8), pathologic surgical debridement (n = 3) or osteomyelitis scans (n = 7) for the diagnosis of sternal osteomyelitis. All patients received primary treatments with debridement and empiric antibiotics treatment, and then guided by antibiotic susceptibility tests. Six of them received additional HBO2 therapy. The others did not received HBO2 therapy because of the insurance or risk of HBO2 therapy. The characteristics of the study population are presented in Table 1 and there was no significant difference between these two groups. The bacteriology data from sternal wound/pus, pleural effusion and blood are listed in Table 2. *Staphylococcus species *were the most common pathogens of sternal wound infection (11/12, 91.7%) and bacteremia (5/6, 83.3%). Mixed infection due to Gram-positive and Gram-negative pathogens was also identified in 2 patients (2/12, 16.7%). Mycobacterium tuberculosis was found in one sternal pus/wound pathogen. HBO2 therapy did not cause any treatment-related complication in the study patients who received this additional treatment. Besides, patients breathed with room air when initiation of HBO2 therapy and did not receive mechanical ventilation, sedative or inotropic medication during HBO2 therapy. Total and average debridement duration, debridement frequency, hospital admission frequency and length of hospital stay were not significantly different between HBO2 therapy and control group. Most importantly, length of ICU stay (8.7 ± 2.7 days vs. 48.8 ± 10.5 days, p < 0.05), duration of invasive mechanical ventilation (MV) (4 ± 1.5 days vs. 34.8 ± 8.3 days, p < 0.05) and duration of noninvasive positive pressure ventilation (NIV) (4 ± 1.9 days vs. 22.3 ± 6.2 days, p < 0.05) were significantly reduced in HBO2 therapy group (Table 3). In addition, hospital death (0 person vs. 3 persons, p < 0.05) w as significantly lower in HBO2 therapy group (Table 4). The causes of death in the control group were ischemic bowel disease (n = 1), acute pancreatitis (n = 1) and mediastinitis (n = 1).

**Table 1:** Characteristiscs of patients in HBO2 and control group

	HBO2(n = 6)	Control(n = 6)	p
Age, years	54.7 ± 7.4	63.3 + 5.5	0.267
Female	1(%17)	2(%34)	0.500
Smoke	3(%50)	3(%50)	0.716
Body mass index (kg/m^2^)	24.7+1.5	24.7+1.5	0.749
Coronary artery disease	3(%50)	3(%83)	0.273
Hypertension	3(%50)	3(%50)	0.716
Atrial fibrillation	1(%17)	1(%17)	0.773
Myocardial infarction history	1(%17)	4(%67)	0.121
Hyperlipidemia	1(%17)	2(%34)	0.500
Congestive heart failure	0	2(%34)	0.227
COPD	1(%17)	1(%17)	0.773
Myasthenia gravis	2(%34)	0	0.227
Diabetes mellitus	1(%17)	3(%50)	0.333
Chronic renal insufficiency	0	1(%17)	0.600
Uremia	1(%17)	1(%17)	0.773

Body mass index: the weight in kilograms divided by the square of the height in meters COPD: chronic obstructive pulmonary disease

**Table 2:** Causative organisms of patients in HBO2 and control group

		HBO2	Control
Sternal pus/wound	Methicillin sensitive *Staphylococcus aureus*	0	1
	Methicillin resistent *Staphylococcus aureus*	5*	4
	Coagulase negative *Staphylococcus species*	0	1*
	*Acinectobacter baumannii*	0	1*
	*Klebsiella pneumoniae*	1*	0
	*Escherichia coli*	1*	0
	*Mycobacterium tuberculosis*	1	0
			
Pleural effusion	Methicillin resistent *Staphylococcus aureus*	1	2
			
Blood	Methicillin resistent *Staphylococcus aureus*	2	3
	*Acinectobacter baumannii*	0	1*
	*Serratia marcescens*	0	1*
	*Proteus mirabilis*	0	1*
	Fungus	0	1*

* mixed infection

**Table 3:** Operative and postoperative details in HBO2 and control group

	HBO2	Control	p
Total debridement operation time (minutes)	437 ± 81.4	355 ± 82.9	0.873
Average debridement operation time (minutes)	94 ± 21.6	98.6 ± 16	0.522
Total debridement frequency	5.5 ± 1.5	3.8 ± 1.0	0.326
APACHE II score	19.7 ± 7.7	18.7 ± 3.7	0.749
Duration in ICU (days)	8.7 ± 2.7	48.8 ± 10.5	0.004
Duration with MV use (days)	4 ± 1.5	34.8 ± 8.3	0.008
Duration with NIV use (days)	4 ± 1.9	22.3 ± 6.2	0.015
HBO2 frequency	21.3 ± 2.5	0	<0.001
Hospital admission frequency	2.8 ± 0.4	2 ± 0.2	0.127
Total hospital length of stay (days)	151 ± 21	138 ± 37	0.757

MV: invasive mechanical ventilation

NI: non-invasive mechanical ventilation

**Table 4:** Outcome of patients in HBO2 and control group

	HBO2	Control	p
Death	0	3	0.046
Complications	0.9 ± 2.3	0.7 ± 5	0.059

Complication: seizure/stroke, acute myocardial infarction/arrhythmia, bacteremia, pneumonia/empyema, acute pancreatitis, ischemic bowel disease, upper gastrointestinal tract bleeding, hyperglycemia, acute renal failure

## Discussion

The incidence of sternal infection and osteomyelitis in patients undergoing sternotomy for cardiothoracic surgery is not common- stated as being less than 4% in several reports; however, it is a serious complication that increases the length of hospital stay, short-term and long term mortality, and medical cost [4,13]. In the current study, we demonstrated that in patients with sternal infection and osteomyelitis after sternotomy and cardiothoracic surgery, HBO2 therapy could be an adjunctive treatment to improve clinical outcomes including the length of ICU stay, duration of MV and NIV support, complications during hospital stay, and hospital mortality.

Risk factors for deep sternal wound infection and osteomyelitis after median sternotomy have been reported that included: (1) preoperative factors: male, chronic obstructive pulmonary disease, diabetes, New York Heart Association congestive heart failure class, reoperation and obesity; (2) intraoperative factors: coronary artery bypass grafting, prolonged cardiopulmonary bypass time and duration of surgery; (3) postoperative factors: excessive postoperative bleeding, postoperative inotropic support and prolonged time (>48 hours) on mechanical ventilation [14-18]. In our study, although patients in the control group compared to HBO2 group had more above-mentioned risk factors, these differences did not reach statistical significance. However, the potential influence by these risk factors might still play a role on the patient outcome. Importantly, patients with the co-morbidity of congestive heart failure have been mentioned the risk for acute pulmonary edema after HBO2 therapy [19]. Therefore, HBO2 therapy should be cautious when be applied to these patients.

The pathophysiology of sternal wound infection and osteomyelitis is hypoxia and ischemia [3,4]; therefore, the administration of HBO2 therapy has theoretical benefit for these patients. However, there are only few case reports and non-randomized studies about the use of HBO2 therapy in this patient population. Recently, Higuchi *et al. *presented 4 patients with sternal osteomyelitis after lung transplantation who received surgical debridement, antimicrobial treatment and adjunctive HBO2 therapy [10]. Three of them improved significantly and the authors concluded that HBO2 therapy was safe and effective for the management of infection complication. Barili *et al *also conducted a prospective nonrandomized study to investigate the effect of HBO2 therapy on organ/space sternal surgical site infection (SSI) following cardiothoracic surgery [20]. A total of 34 patients who developed organ/space sternal SSI after cardiac surgery were enrolled and divided into two groups according to whether HBO2 therapy was offered or not. The relapsing, infection rate, the duration of intravenous antibiotic use and total hospital stay were significantly lower in patients with HBO2 therapy than those without HBO2 therapy. In our study, we further identified that HBO2 therapy alleviated the burden of critical care by shortening the length of ICU stay, and duration of MV and NIV support. Furthermore, it also reduced complications during hospital stay and mortality in patients with sternal infection and osteomyelitis after sternotomy and cardiothoracic surgery. Although the total length of hospital stay and total debridement frequency were not significantly different between the HBO2 therapy and control group, patients in HBO2 group tended to receive more debridement frequencies and have more hospital admissions that might contribute to longer length hospital stay and better outcome.

The current treatment for sternal infection and osteomyelitis includes early recognition, early debridement, collection of specimens for bacteria pathogen, use of broad-spectrum antibiotics and a change in antibiotics based on the culture result of the sensitivity test. When the patient's infection is under control, reconstruction of the wound should be instituted, including rewiring, and pectoralis or rectus muscle and omental flap rotation [21-23]. HBO2 therapy might offer additional biochemical and cellular effects. With the administration of HBO2 therapy, the partial oxygen pressure in the wound increases and promotes collagen matrix formation, angiogenesis, osteoclast/osteoblast activity and bone union [24-26]. Increased oxygen tension improves neutrophil ability to kill bacteria. Some antibiotics such as aminoglycosides, fluoroquinolones, vancomycin and sulfonamides have a synergistic effect when combined with HBO2 for the treatment of bacterial infection [9]. HBO2 itself also acts as an antibiotic agent against a broad spectrum of Gram-positive and Gram-negative bacteria [9]. These above-mentioned effects of HBO2 therapy might explain how adjunctive HBO2 therapy provides additional benefits to patients with sternal infection and osteomyelitis and improves the clinical outcomes of such patients.

The timing of HBO2 therapy for SSI and sternal osteomyelitis after sternotomy has been discussed. Higuchi *et al. *reported the initiation of HBO2 therapy for patients with persistent osteomyelitis after standard treatment [10]. Barili *et al *started early HBO2 therapy after the diagnosis of sternal surgical site infection [20]. Both studies demonstrated the adjunctive benefit of HBO2 therapy. In our study, the decision of HBO2 therapy was based on clinical physicians, range from 2 weeks to months after the diagnosis of sternal osteomyelitis. However, the timing of HBO2 therapy for sternal osteomyelitis to gain maximum benefit requires further investigation.

Although the microbiologic pathogens from the sternal pus/wound, pleural effusion and blood are diverse, Gram-positive cocci remain the most common pathogens in our retrospective study which is consistent with other studies [27,28]. Interesting, mycobacterium tuberculosis was found in one sternal pus/wound pathogen. Reactivation of pulmonary or mediastinal lymph node tuberculosis, contamination of the operation field, and exogenous exposure persons with active pulmonary tuberculosis may hypothesize tuberculosis infection after cardiothoracic surgery [29,30]. The patient with sternal wound infection of mycobacterium tuberculosis received surgical debridement, combination antituberculous agent treatment and HBO2 therapy and was finally discharged. It is important to note that some of the patients with sternal infection and osteomyelitis developed bacteremia (2/6 in HBO2 therapy group, 4/6 in control group) which might worsen the prognosis of patients. Based on our observations of this study, HBO2 therapy might improve sternal infection and reduce the occurrence of bacteremia.

## Limitations

Our study had several limitations that are worth noting. This study was retrospective in nature; therefore certain data might have been missing or poorly documented. Second, the number of patients in this study was small. Third, we only focused on the clinical outcomes of the study patients; therefore, the local effects of HBO2 therapy, such as tissue oxygenation, angiogenesis, and osteoclast/osteoblast activity were not observed.

## Conclusion

HBO2 counteracts tissue hypoxia by elevating tissue oxygen partial pressure, promotes wound healing, has the synergistic effect when combined with some antibiotics and prevents infection. In this study, all patients who received HBO2 therapy as an adjunctive treatment for post-cardiothoracic surgery related sternal infection and osteomyelitis achieved a favorable result. We suggest a combination of aggressive surgical debridement, antibiotic treatment and adjunctive HBO2 therapy for patients who develop sternal infection and osteomyelitis after cardiothoracic surgery.

## Competing interests

The authors declare that they have no competing interests.

## Authors' contributions

WKY and YWC both participated in the design of the study and drafted the manuscript. HGS and TCL both obtained data and performed the statistical analysis. HKK and JHW both participated in critical revision of the manuscript. All authors read and approved the final manuscript
